# Nomogram basing pre-treatment parameters predicting early response for locally advanced rectal cancer with neoadjuvant chemotherapy alone: a subgroup efficacy analysis of FOWARC study

**DOI:** 10.18632/oncotarget.6469

**Published:** 2015-12-04

**Authors:** Jianwei Zhang, Yue Cai, Huabin Hu, Ping Lan, Lei Wang, Meijin Huang, Liang Kang, Xiaojian Wu, Hui Wang, Jiayu Ling, Jian Xiao, Jianping Wang, Yanhong Deng

**Affiliations:** ^1^ Department of Medical Oncology, The Sixth Affiliated Hospital of Sun-Yat Sen University, Guangzhou, Guangdong, P.R. China; ^2^ Department of Colorectal Surgery, The Sixth Affiliated Hospital of Sun-Yat sen University, Guangzhou, Guangdong, P.R. China

**Keywords:** neoadjuvant chemotherapy, nomogram, predictive, rectal cancer

## Abstract

**Objective:**

To develop an accurate model with pre-treatment parameters to predict tumor regression and down-staging in locally advanced rectal cancer patients, basing the cohort of preoperative chemotherapy alone in FOWARC study.

**Patients and Methods:**

From Jan 2011 to Feb 2015, complete data was available for 137 out of 165 patients who received preoperative chemotherapy alone. All pre-treatment clinical parameters were collected. Tumor regression grade (TRG) 0-1 was defined as good regression, and pathological TNM stage (ypTNM) 0-I after neoadjuvant treatment was defined as good down-staging. Nomogram was established to predict tumor regression and down-staging. The predictive performance of the model was assessed with concordance index and calibration plots.

**Results:**

Of the 137 patients, 10 had TRG 0 (complete regression); 32 patients, TRG 1; and 95 patients, TRG 2 and 3 (poor regression); 56 (40.9%) patients were classified as good down-staging with ypTNM stage 0-I. The predictive nomograms were developed to predict the probability of TRG 0-1 and good down-staging with a C-index of 0.72 (95% CI: 0.604-0.797) and 0.76 (95% CI: 0.681-0.844). Calibration plots showed good statistical performance on internal validation. Predictive factors in the models included tumor length, tumor circumferential extent, age, and ApoA1.

**Conclusions:**

The model based on available clinical parameters could accurately predict early efficacy with neoadjuvant mFOLFOX6 chemotherapy alone, which might help in patient selection for optimized treatment.

## INTRODUCTION

In the past few decades, treatment outcomes for rectal cancer have shown tremendous improvement. Adoption of better surgical techniques and total mesorectal excision (TME) are cornerstones of the therapy [1, 2]. The introduction of neoadjuvant treatment in locally advanced rectal cancer further decreased the risk of local-regional recurrence [3, 4]. After publication of the CAO/ARO/AIO-94 (Working Group of Surgical Oncology/Radiation Oncology/Medical Oncology of the German Cancer Society) study in 2004 [5], preoperative chemoradiotherapy (CRT) with infusional fluorouracil and TME surgery became the standard-of-care for patients with stage II-III rectal cancer. The incidence of local recurrence at 5 and 10 years in preoperative CRT group was 5% and 7.1%, respectively, while in postoperative CRT group, the incidence was 9.7% and 10.1% [6]. However, about 30% of patients still developed distant metastasis after long-term follow-up, which remains the main obstacle for improving survival. The survival of patients with LARC is still as low as 65% [6]. Hence more effective systemic treatment options are needed.

On the other hand, with the modern TME-based surgical techniques, the local recurrence rate is under control [7]. In the CAO/ARO/AIO-94 study, only about 3% of patients benefited from preoperative CRT [6]. Besides, preoperative radiation led to anal and sexual functional concerns with no survival benefit [8]. The major challenge that needs to be addressed immediately is that rectal cancer patients do not undergo selective preoperative radiotherapy.

Neoadjuvant chemotherapy-alone approach has been proposed [9]. A prospective pilot study by investigators of Memorial Sloan-Kettering Cancer Center evaluated neoadjuvant chemotherapy alone, with six cycles of FOLFOX plus bevacizumab in locally advanced rectal cancer patients. Thirty-two patients were enrolled with the pathological complete response (pCR) rate of 25% [10]. The promising results suggested that in the era of TME and high-quality magnetic resonance imaging (MRI), radiotherapy might be omitted in selected patients.

To avoid the damage of radiotherapy for rectal cancer patients, we designed a phase II study comparing neoadjuvant FOLFOX6 chemotherapy with or without radiation in locally advanced rectal cancer. The preliminary results were reported in ASCO annual meeting 2015 [11]. In the group of patients with neoadjuvant mFOLFOX6 chemotherapy alone, about 40% of patients showed good down-staging, and radiation therapy should be avoided in these chemo-sensitive patients.

In the past, many studies tried to find biomarkers to predict prognosis of rectal cancer after preoperative CRT, such as epithelial growth factor receptor (EGFR) and thymidylate synthase (TS) genes, and carcinoembryonic antigen [12-15]. However, most of them have not been used in routine clinical practice. Some other studies have established a nomogram predictive model with sequential PET-CT imaging for rectal cancer in patients receiving preoperative CRT, which is expensive and was also not regularly recommended in the management of rectal cancer [16, 17]. Until now, no predictive model has been developed for preoperative chemotherapy alone in locally advanced rectal cancer.

In this prospective study, we aimed to develop an accurate model and nomogram with available pre-treatment parameters to predict efficacy of locally advanced rectal cancer with mFOLFOX6 chemotherapy alone.

## MATERIALS AND METHODS

### Patients

From Jan 2011 to Feb 2015, a total of 165 patients were enrolled into the group receiving preoperative mFOLFOX6 chemotherapy alone, among which, complete information was available in 137 patients. All patients were >18 years with histopathologically confirmed rectal cancer with an inferior margin no more than 12 cm above the anal verge assessed by MRI or CT with rectal ultrasound. The clinical stage was cT 3/4 or lymph node involvement (cN+) without distant metastasis as assessed by MRI or multislice CT. MRI was recommended for local staging. All patients were treated according to the protocol (NCT01211210) with 4 cycles of mFOLFOX6 chemotherapy alone before surgery and then underwent TME resection.

All the available pre-treatment clinical parameters were collected, including gender, age, clinical TNM stage, tumor length, tumor circumferential extent, and distance from the tumor inferior margin to the anal verge. The blood biomarkers including blood routine test (white blood cell count [WBC], hemoglobin, lymphocyte, neutrophil, and monocyte), blood biochemistry (lactate dehydrogenase [LDH], alanine transaminase [ALT], aspartate aminotransferase [AST], total bilirubin, direct bilirubin, serum creatinine, cholesterol, high-density lipoprotein, low-density lipoprotein, triglyceride, apolipoprotein A-1 [ApoA1], and apolipoprotein B) and serum tumor markers (carcinoembryonic antigen and carbohydrate antigen 19-9) were also analyzed.

### Pathological assessment

All resection specimens were assessed by two pathologists blinded to clinical outcomes of the patients, according to American Joint Committee on Cancer (AJCC) TNM staging category (y prefix indicated classification after neoadjuvant treatment). Tumor stage grouping, numbers of examined and involved lymph nodes were recorded.

Pathologic tumor regression grade (TRG) was assessed semi-quantitatively by determining the amount of viable tumor versus fibrotic tissue according to the College of American Pathologists' guidelines [18]. Tumor response was graded on a scale of 0 (complete response; no viable cancer cells) to 3 (poor response; minimal or no regression, extensive residual cancer), wherein grade 1 referred to moderate response or minimal residual cancer, and grade 2 indicated minimal response. In this study, TRG 0-1 was defined as good regression, while tumor down-staging to ypT_0-2_N_0_M_0_ was defined as good down-staging.

All patients gave written informed consent before entering the study. The study was approved by local medical ethics committee and was conducted in accordance to the Declaration of Helsinki and good clinical practice.

### Statistical analysis

Binary logistic regression was used to analyze the effect of all parameters on TRG and down-staging. Variables that achieved significance at *P* < 0.05 were entered into the multivariable analyses via the logistic regression model. And the parameters that were significant under clinical consideration were also incorporated into the model. Statistical analyses to identify independent prognostic factors were conducted in SPSS 16.0 for Windows (SPSS, Chicago, IL). On the basis of the results of the multivariable analysis, a nomogram was formulated to provide visualized probability prediction using R 2.13.1 (http://www.r-project.org) with the survival and rms package.

### Calibration and internal validation of the nomogram

The nomogram was validated internally with 1000 bootstrap resamples. The model performance for predicting outcome was evaluated by calculating the concordance index (C-index). The value of the C-index ranges from 0.5 to 1.0, with 0.5 indicating a random chance and 1.0 indicating a perfect ability to correctly discriminate the outcome with the model. Calibration of the nomogram for TRG and down-staging were performed by comparing the predicted survival with the observed survival after bias correction.

## RESULTS

### Clinicopathologic characteristics of patients

Of the 137 patients, the median age was 57 years (range: 22 to 75 years). Most patients were men (70.8% *vs*. 29.2%), and most were stage III at first diagnosis. Table 1 lists the clinicopathologic characteristics of all patients. Among the collected variables, lactate dehydrogenase was not considered for analysis due to the high rate of missing values. Tumor length and the distance from the anal verge were detected with MRI. Tumor length was the distance from the inferior margin to the superior margin of the tumor. The extent of tumor circumference was defined as the quartiles of luminal circumference measured by endoscope or MRI.

**Table 1:** Patients basic characteristics

Parameters	N (%)
**Age (years)**	
** Median**	57
** Range**	(22-75)
**Gender**	
** Male**	97 (70.8%)
** Female**	40 (29.2%)
**Clinical Tumor stage (cT)**	
** T_2_**	2 (1.5%)
** T_3_**	120 (87.6%)
** T_4_**	15 (10.9%)
**Clinical Nodal stage (cN)**	
** N_0_**	43 (31.4%)
** N_1_**	56 (40.9%)
** N_2_**	38 (27.7%)
**cTNM staging**	
** Stage II**	40 (34.5%)
** Stage III**	76 (65.5%)
**CEA**	
** Median**	2.38
** Range**	0.5-56.9
**Tumor length (cm)**	
** Median**	4.3
** Range**	(1.2-10)
**Distance from anal verge (cm)**	
** Median**	6
** Range**	1.4-12
**Tumor circumferential extent**	
** Median**	0.75
** Range**	(0.25-1)
**Tumor regression grade (TRG)**	
** 0-1**	42 (30.7%)
** 2-3**	95 (69.3%)
**Good downstaging (ypT0-2N0)**	
** Yes**	56 (40.9%)
** No**	81 (59.1%)

CEA, carcinoembryonic antigen

With pathological assessment, 10 patients had achieved TRG 0 (complete regression), 32 patients with TRG 1, and 95 patients with TRG 2 and 3 (poor regression); 56 (40.9%) patients were classified as having good down-staging with ypT0-2N0 (stage 0 and stage I). No patients developed distant metastasis immediately after neoadjuvant chemotherapy or surgery.

### Independent predictive factors in the study cohort

Univariate analysis was performed on all the variables. Table 2 lists the results. Tumor length, tumor circumferential extent, and ApoA1 were predictive factors for good tumor regression (TRG 0-1) and good down-staging, whereas young age was only associated with good down-staging. Surprisingly, clinical tumor stage and clinical lymph node stage were not correlated with early efficacy. Other parameters, including gender, distance from anal verge, tumor marker (CEA, CA19-9), and blood routine test were all not significantly different.

**Table 2:** Univariate and multivariate analysis of pre-treatment parameters

Prarameters	Univariate analysis		Multivariate analysis				
	TRG 0-1	ypT0-2N0	TRG 0-1		ypT_0-2_N_0_		
	*P*	*P*	OR (95% CI)	*P*	OR(95% CI)	*P*	
**Age**	0.39	0.006*			0.08 (0.02-0.38)	0.02*	
**Gender**	0.91	0.50					
**Tumor length**	0.007*	0.004*	0.98 (0.95-1.01)	0.24	0.98 (0.96-1.01)	0.075	
**Tumor circumferential extent**	0.004*	0.002*	0.11 (0.01-0.44)	0.014*	0.09 (0.02-0.47)	0.004*	
**Distance from anal verge**	0.51	0.46					
**cT**	0.16	0.84					
**cN**	0.91	0.19					
**CEA**	0.63	0.54					
**CA 19-9**	0.21	0.74					
**ApoA1**	0.01*	0.02*	7.51 (1.11-50.8)	0.038*	3.57 (0.53-23.9)	0.056	
**ApoB**	0.49	0.54					
**ALT**	0.89	0.95					
**AST**	0.48	0.59					
**TBiL**	0.29	0.30					
**ALB**	0.31	0.62					
**WBC**	0.64	0.70					
**Hemoglobin**	0.06	0.34					
**Lymphoctye**	0.64	0.60					

WBC, white blood cell count; CEA, carcinoembryonic antigen; CA19-9, carbohydrate antigen 19-9; ApoA1, Apolipoprotein A-1; ApoB, Apolipoprotein B; ALT, alanine transarninase; AST, aspartate aminotransferase; TBiL, total bilirubin; ALB, albumin

All significant parameters in the univariate analysis were entered into the multivariable analysis based on the logistic regression (Table 2). Only tumor circumferential extent (*P* = 0.014) and ApoA1 (*P* = 0.038) were independent predictors for TRG 0-1, while for good down-staging, only age (*P* = 0.02) and tumor circumferential extent (*P* = 0.004) were independent predictors. Tumor length has been reported earlier as an important predictor for pCR when receiving preoperative chemo-radiation [16]. Here we also selected tumor length into the model. ApoA1 was also included due to significance near decision boundary (*P* = 0.056).

Hence, the final selected predictors in the multivariate model were age, tumor length, tumor circumferential extent, and ApoA1.

### Predictive nomograms established for early efficacy

**Figure 1:**
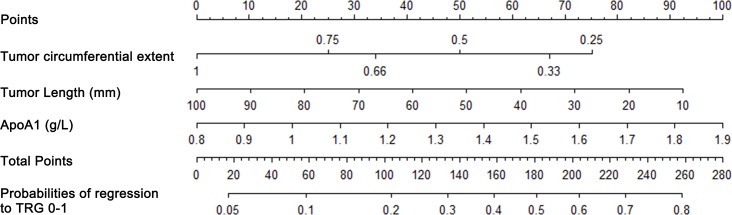
Nomogram for good regression prediction A score for each predictor can be read out at the top scale (score). All summed scores can be converted directly to the probability of response.

**Figure 2:**
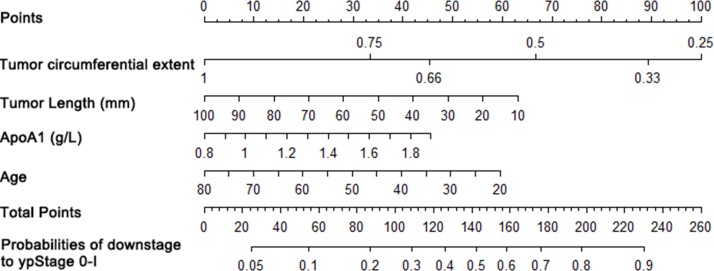
Nomogram for good down-staging prediction A score for each predictor can be read out at the top scale (score). All summed scores can be converted directly to the probability of response.

Nomograms that incorporated the selected predictive factors were established (Figures 1 and 2). The nomogram demonstrated that tumor length and ApoA1 shared the largest contribution to good regression, followed by tumor circumferential extent. In predicting good down-staging, tumor circumferential extent showed the greatest contribution, followed by tumor length, Age, and ApoA1. Each of these variables was assigned a score on the point scale. Through adding up of the score of each variable and referring to the total point scale, we could draw a straight line to determine the estimated probability of TRG 0-1 and ypT_0-2_N_0_.

### Calibration of the nomogram

**Figure 3:**
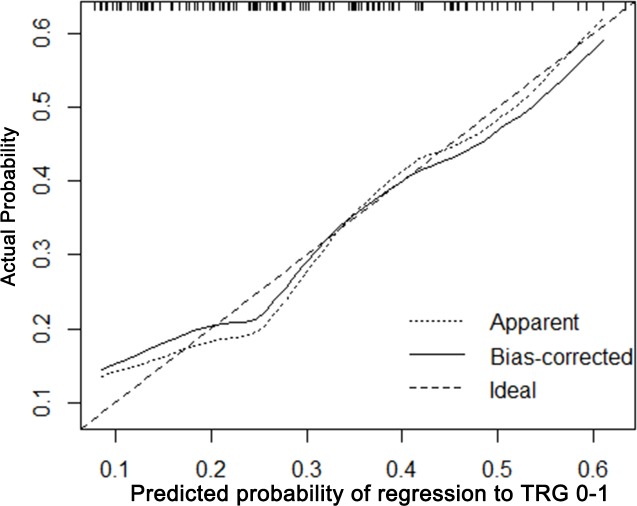
Calibration plot of the predicted and observed probabilities of regression to TRG 0-1 The prediction calculated using the nomograms were plotted on the X-axis, and the observed rate of regression and down-staging is plotted on the Y-axis.

**Figure 4:**
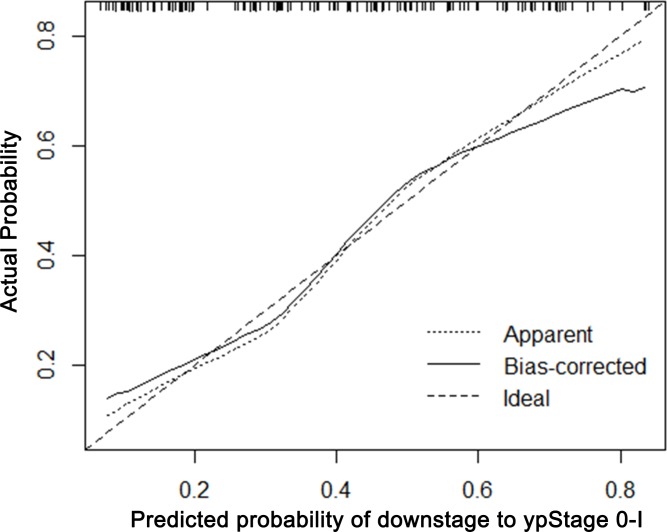
Calibration plot of the predicted and observed probabilities of regression to down-staging to ypStage 0-I The prediction calculated using the nomograms were plotted on the X-axis, and the observed rate of regression and down-staging is plotted on the Y-axis.

The calibration plots presented good statistical performance upon internal validation between the nomogram prediction and actual observation for probability of TRG 0-1 (Figure 3) and good down-staging (Figure 4). The Harrell's concordance index (C-index) for the established nomogram to predict tumor regression to TRG 0-1 was 0.72 (95% CI: 0.604-0.797) and 0.762 (95% CI: 0.681-0.844) for good down-staging.

## DISCUSSION

Chemotherapy-alone approach showed promising efficacy in the preliminary results of FOWARC study, although it is not the standard-of-care for locally advanced rectal cancer. [11]. To the best of our knowledge, this is the first nomogram to predict the early efficacy of neoadjuvant chemotherapy alone in locally advanced rectal cancer with a prospective cohort. Previously, all the predictive models were established basing on chemoradiotherapy [16, 17, 19].

As is known, pCR is the most robust surrogate endpoint for early efficacy and long-term survival in locally advanced rectal cancer [20]. However, in this prospective cohort, pCR rate was only about 7%, which is too low to build a model. Hence we measured the early efficacy in two ways, TRG 0-1 (30.6%) and ypT_0-2_N_0_(40.9%) in this study. In fact, the most frequently used method to distinguish responders from non-responders is by means of TRG [18]. Furthermore, it has been demonstrated that TRG is the independent prognostic factor for cumulative incidence of distant metastasis and disease-free survival after CRT [21].

All the predictors in the nomogram were available in routine pre-treatment examination, which was easily to collect. Tumor length and tumor circumferential extent were selected in the model, both referred to tumor load. Tumor length has previously been reported to be the most important predictor for pCR in rectal cancer after neoadjuvant CRT [16]. In multivariate analysis, tumor length was not a predictor. Given clinical consideration, we still integrated it into the model, which increased the c-index of the predictive model. As for tumor circumferential extent, it has been proven that circumferential extent of tumors less than 1/2 cycle is associated with higher pCR rate [22]. Overall, tumor dimension is one of the important predictive factors for tumor regression and tumor down-staging.

Interestingly, ApoA1 showed predictive value in the analysis. In fact, the relationship between ApoA1 and tumorigenesis and development had been investigated in the past few years. It has been reported that the serum concentration of ApoA1 in breast cancer, colorectal cancer, and pancreatic cancer was lower than that of the control group, which was considered to be a serum marker for detecting carcinoma [23-25]. In preclinical study, ApoA1 has shown the potency of anti-angiogenesis [26]. Besides, in breast cancer, the expression level of ApoA1 increased in patients who showed good response to preoperative chemotherapy, which demonstrated that higher ApoA1 increased chemo-sensitivity [23, 27-29]. Here in the current study, we found that higher ApoA1 level was related to better response. Further investigations are warranted on the detailed mechanism of ApoA1 in increasing the efficacy of chemotherapy.

Age is one of the predictor in tumor down-staging. Young age was associated with better efficacy. Vincenzo, et al. also built a nomogram for predicting overall survival in rectal cancer. Age was also a positive predictor in the model. [30]. Surprisingly, CEA was not a predictive factor in our model. It has been reported that lower CEA level was correlated with better efficacy and long-term survival in rectal cancer after neoadjuvant CRT [12, 17, 31-34]. In our study, serum CEA level was lower than 5 ng/ml in most of the patients, with a median value of 2.38 ng/ml, which might contribute to the negative results. All the parameters were collected before administering any treatment. We only collected available clinical parameters. More predictors could be added to increase the performance of the model, including imaging variables such as apparent diffusion coefficient or T2 mapping of MRI [35-37] and biological variables such as gene signatures [15].

The limitations of the study are as follows. First of all, the samples size is relatively small. Second, external validation was not performed due to lack of external cohort, because neoadjuvant chemotherapy alone is still not the standard care for locally advanced rectal cancer. Prospective validation of the model is still required to ensure sufficient statistical power for clinical application.

In conclusion, the novel nomogram for predicting early efficacy of patients with locally advanced rectal cancer after receiving neoadjuvant mFOLFOX6 chemotherapy alone might promote personalized treatment approach in the future.

## References

[1] (2001). Adjuvant radiotherapy for rectal cancer: a systematic overview of 8,507 patients from 22 randomised trials. LANCET.

[2] Valentini V, Beets-Tan R, Borras JM, Krivokapic Z, Leer JW, Pahlman L, Rodel C, Schmoll HJ, Scott N, Velde CV, Verfaillie C (2008). Evidence and research in rectal cancer. RADIOTHER ONCOL.

[3] Valentini V, Aristei C, Glimelius B, Minsky BD, Beets-Tan R, Borras JM, Haustermans K, Maingon P, Overgaard J, Pahlman L, Quirke P, Schmoll HJ, Sebag-Montefiore D, Taylor I, Van Cutsem E, Van de Velde C (2009). Multidisciplinary Rectal Cancer Management: 2nd European Rectal Cancer Consensus Conference (EURECA-CC2). RADIOTHER ONCOL.

[4] Schrag D (2013). Evolving role of neoadjuvant therapy in rectal cancer. Curr Treat Options Oncol.

[5] Sauer R, Becker H, Hohenberger W, Rodel C, Wittekind C, Fietkau R, Martus P, Tschmelitsch J, Hager E, Hess CF, Karstens JH, Liersch T, Schmidberger H, Raab R (2004). Preoperative versus postoperative chemoradiotherapy for rectal cancer. N Engl J Med.

[6] Sauer R, Liersch T, Merkel S, Fietkau R, Hohenberger W, Hess C, Becker H, Raab HR, Villanueva MT, Witzigmann H, Wittekind C, Beissbarth T, Rodel C (2012). Preoperative versus postoperative chemoradiotherapy for locally advanced rectal cancer: results of the German CAO/ARO/AIO-94 randomized phase III trial after a median follow-up of 11 years. J CLIN ONCOL.

[7] Quirke P, Steele R, Monson J, Grieve R, Khanna S, Couture J, O'Callaghan C, Myint AS, Bessell E, Thompson LC, Parmar M, Stephens RJ, Sebag-Montefiore D (2009). Effect of the plane of surgery achieved on local recurrence in patients with operable rectal cancer: a prospective study using data from the MRC CR07 and NCIC-CTG CO16 randomised clinical trial. LANCET.

[8] Peeters KC, van de Velde CJ, Leer JW, Martijn H, Junggeburt JM, Kranenbarg EK, Steup WH, Wiggers T, Rutten HJ, Marijnen CA (2005). Late side effects of short-course preoperative radiotherapy combined with total mesorectal excision for rectal cancer: increased bowel dysfunction in irradiated patients—a Dutch colorectal cancer group study. J CLIN ONCOL.

[9] Cercek A, Weiser MR, Goodman KA, Reidy DL, Wong WD, Guillem JG, Temple LK, Schrag D, Paty P, Saltz L (2010). Complete pathologic response in the primary of rectal or colon cancer treated with FOLFOX without radiation. J Clin Oncol.

[10] Schrag D, Weiser MR, Goodman KA, Gonen M, Hollywood E, Cercek A, Reidy-Lagunes DL, Gollub MJ, Shia J, Guillem JG, Temple LK, Paty PB, Saltz LB (2014). Neoadjuvant chemotherapy without routine use of radiation therapy for patients with locally advanced rectal cancer: a pilot trial. J CLIN ONCOL.

[11] Yanhong Deng, Pan Chi Ping Lan (2015). A multi-center randomized controlled trial of mFOLFOX6 with or without radiation in neoadjuvant treatment of local advanced rectal cancer (FOWARC study): Preliminary results. J Clin Oncol.

[12] Wallin U, Rothenberger D, Lowry A, Luepker R, Mellgren A (2013). CEA - a predictor for pathologic complete response after neoadjuvant therapy for rectal cancer. DIS COLON RECTUM.

[13] Kleiman A, Al-Khamis A, Farsi A, Kezouh A, Vuong T, Gordon PH, Vasilevsky CA, Morin N, Faria J, Ghitulescu G, Boutros M (2015). Normalization of CEA Levels Post-Neoadjuvant Therapy is a Strong Predictor of Pathologic Complete Response in Rectal Cancer. J GASTROINTEST SURG.

[14] Spolverato G, Pucciarelli S, Bertorelle R, De Rossi A, Nitti D (2011). Predictive factors of the response of rectal cancer to neoadjuvant radiochemotherapy. Cancers (Basel).

[15] Agostini M, Crotti S, Bedin C, Cecchin E, Maretto I, D'Angelo E, Pucciarelli S, Nitti D (2014). Predictive response biomarkers in rectal cancer neoadjuvant treatment. Front Biosci (Schol Ed).

[16] van Stiphout RG, Lammering G, Buijsen J, Janssen MH, Gambacorta MA, Slagmolen P, Lambrecht M, Rubello D, Gava M, Giordano A, Postma EO, Haustermans K, Capirci C, Valentini V, Lambin P (2011). Development and external validation of a predictive model for pathological complete response of rectal cancer patients including sequential PET-CT imaging. RADIOTHER ONCOL.

[17] Buijsen J, van Stiphout RG, Menheere PP, Lammering G, Lambin P (2014). Blood biomarkers are helpful in the prediction of response to chemoradiation in rectal cancer: a prospective, hypothesis driven study on patients with locally advanced rectal cancer. RADIOTHER ONCOL.

[18] Washington MK, Berlin J, Branton P, Burgart LJ, Carter DK, Fitzgibbons PL, Halling K, Frankel W, Jessup J, Kakar S, Minsky B, Nakhleh R, Compton CC (2009). Protocol for the examination of specimens from patients with primary carcinoma of the colon and rectum. ARCH PATHOL LAB MED.

[19] van Stiphout RG, Valentini V, Buijsen J, Lammering G, Meldolesi E, van Soest J, Leccisotti L, Giordano A, Gambacorta MA, Dekker A, Lambin P (2014). Nomogram predicting response after chemoradiotherapy in rectal cancer using sequential PETCT imaging: a multicentric prospective study with external validation. RADIOTHER ONCOL.

[20] Maas M, Nelemans PJ, Valentini V, Das P, Rodel C, Kuo LJ, Calvo FA, Garcia-Aguilar J, Glynne-Jones R, Haustermans K, Mohiuddin M, Pucciarelli S, Small WJ, Suarez J, Theodoropoulos G, Biondo S (2010). Long-term outcome in patients with a pathological complete response after chemoradiation for rectal cancer: a pooled analysis of individual patient data. LANCET ONCOL.

[21] Fokas E, Liersch T, Fietkau R, Hohenberger W, Beissbarth T, Hess C, Becker H, Ghadimi M, Mrak K, Merkel S, Raab HR, Sauer R, Wittekind C, Rodel C (2014). Tumor regression grading after preoperative chemoradiotherapy for locally advanced rectal carcinoma revisited: updated results of the CAO/ARO/AIO-94 trial. J CLIN ONCOL.

[22] Sun Y, Chi P, Xu B, Lin H, Lu X, Huang Y, Xu Z, Huang S, Jiang C (2014). Predictive factors associated with pathologic complete response after neoadjuvant chemoradiotherapy in rectal cancer [Article in Chinese]. Zhonghua Wei Chang Wai Ke Za Zhi.

[23] Huang HL, Stasyk T, Morandell S, Dieplinger H, Falkensammer G, Griesmacher A, Mogg M, Schreiber M, Feuerstein I, Huck CW, Stecher G, Bonn GK, Huber LA (2006). Biomarker discovery in breast cancer serum using 2-D differential gel electrophoresis/MALDI-TOF/TOF and data validation by routine clinical assays. ELECTROPHORESIS.

[24] Ehmann M, Felix K, Hartmann D, Schnolzer M, Nees M, Vorderwulbecke S, Bogumil R, Buchler MW, Friess H (2007). Identification of potential markers for the detection of pancreatic cancer through comparative serum protein expression profiling. PANCREAS.

[25] Engwegen JY, Helgason HH, Cats A, Harris N, Bonfrer JM, Schellens JH, Beijnen JH (2006). Identification of serum proteins discriminating colorectal cancer patients and healthy controls using surface-enhanced laser desorption ionisation-time of flight mass spectrometry. World J Gastroenterol.

[26] Gao F, Vasquez SX, Su F, Roberts S, Shah N, Grijalva V, Imaizumi S, Chattopadhyay A, Ganapathy E, Meriwether D, Johnston B, Anantharamaiah GM, Navab M, Fogelman AM, Reddy ST, Farias-Eisner R (2011). L-5F, an apolipoprotein A-I mimetic, inhibits tumor angiogenesis by suppressing VEGF/basic FGF signaling pathways. Integr Biol (Camb).

[27] Lane DM, Boatman KK, McConathy WJ (1995). Serum lipids and apolipoproteins in women with breast masses. Breast Cancer Res Treat.

[28] Yiu CC, Sasano H, Ono K, Chow LW (2010). Changes in protein expression after neoadjuvant use of aromatase inhibitors in primary breast cancer: a proteomic approach to search for potential biomarkers to predict response or resistance. Expert Opin Investig Drugs.

[29] Mazouni C, Baggerly K, Hawke D, Tsavachidis S, Andre F, Buzdar AU, Martin PM, Kobayashi R, Pusztai L (2010). Evaluation of changes in serum protein profiles during neoadjuvant chemotherapy in HER2-positive breast cancer using an LC-MALDI-TOF/MS procedure. PROTEOMICS.

[30] Valentini V, van Stiphout RG, Lammering G, Gambacorta MA, Barba MC, Bebenek M, Bonnetain F, Bosset JF, Bujko K, Cionini L, Gerard JP, Rodel C, Sainato A, Sauer R, Minsky BD, Collette L (2011). Nomograms for predicting local recurrence, distant metastases, and overall survival for patients with locally advanced rectal cancer on the basis of European randomized clinical trials. J CLIN ONCOL.

[31] Park YA, Sohn SK, Seong J, Baik SH, Lee KY, Kim NK, Cho CW (2006). Serum CEA as a predictor for the response to preoperative chemoradiation in rectal cancer. J SURG ONCOL.

[32] Moreno GV, Cejas P, Blanco CM, Feliu BJ, de Castro CJ, Belda-Iniesta C, Barriuso J, Sanchez JJ, Larrauri J, Gonzalez-Baron M, Casado E (2009). Prognostic value of carcinoembryonic antigen level in rectal cancer treated with neoadjuvant chemoradiotherapy. INT J COLORECTAL DIS.

[33] Perez RO, Sao JG, Habr-Gama A, Kiss D, Proscurshim I, Campos FG, Gama-Rodrigues JJ, Cecconello I (2009). The role of carcinoembriogenic antigen in predicting response and survival to neoadjuvant chemoradiotherapy for distal rectal cancer. DIS COLON RECTUM.

[34] Giessen C, Nagel D, Glas M, Spelsberg F, Lau-Werner U, Modest DP, Michl M, Heinemann V, Stieber P, Schulz C (2014). Evaluation of preoperative serum markers for individual patient prognosis in stage I-III rectal cancer. Tumour Biol.

[35] Dzik-Jurasz A, Domenig C, George M, Wolber J, Padhani A, Brown G, Doran S (2002). Diffusion MRI for prediction of response of rectal cancer to chemoradiation. LANCET.

[36] Kuroki Y, Nasu K, Kuroki S, Murakami K, Hayashi T, Sekiguchi R, Nawano S (2004). Diffusion-weighted imaging of breast cancer with the sensitivity encoding technique: analysis of the apparent diffusion coefficient value. MAGN RESON MED SCI.

[37] Chen B, Roeder E, Vuissoz PA, Gillet P, Felblinger J, Beaumont M, Pinzano A (2013). Respective interest of T2 mapping and diffusion tensor imaging in assessing porcine knee cartilage with MR at 3 Teslas. Biomed Mater Eng.

